# Mycobacterial Control of Host Mitochondria: Bioenergetic and Metabolic Changes Shaping Cell Fate and Infection Outcome

**DOI:** 10.3389/fcimb.2020.00457

**Published:** 2020-09-30

**Authors:** Krishnaveni Mohareer, Jayashankar Medikonda, Govinda Raju Vadankula, Sharmistha Banerjee

**Affiliations:** Laboratory of Molecular Pathogenesis, Department of Biochemistry, School of Life Sciences, University of Hyderabad, Hyderabad, India

**Keywords:** *Mycobacterium tuberculosis*, bioenergetics, mitochondria, immuno-metabolism, macrophage response, M1, M2

## Abstract

Mitochondria, are undoubtedly critical organelle of a eukaryotic cell, which provide energy and offer a platform for most of the cellular signaling pathways that decide cell fate. The role of mitochondria in immune-metabolism is now emerging as a crucial process governing several pathological states, including infection, cancer, and diabetes. Mitochondria have therefore been a vulnerable target for several bacterial and viral pathogens to control host machinery for their survival, replication, and dissemination. *Mycobacterium tuberculosis*, a highly successful human pathogen, persists inside alveolar macrophages at the primary infection site, applying several strategies to circumvent macrophage defenses, including control of host mitochondria. The infection *perse* and specific mycobacterial factors that enter the host mitochondrial milieu perturb mitochondrial dynamics and function by disturbing mitochondrial membrane potential, shifting bioenergetics parameters such as ATP and ROS, orienting the host cell fate and thereby infection outcome. In the present review, we attempt to integrate the available information and emerging dogmas to get a holistic view of *Mycobacterium tuberculosis* infection vis-a-vis mycobacterial factors that target host mitochondria and changes therein in terms of morphology, dynamics, proteomic, and bioenergetic alterations that lead to a differential cell fate and immune response determining the disease outcome. We also discuss critical host factors and processes that are overturned by *Mycobacterium tuberculosis*, such as cAMP-mediated signaling, redox homeostasis, and lipid droplet formation. Further, we also present alternate dogmas as well as the gaps and limitations in understanding some of the present research areas, which can be further explored by understanding some critical processes during *Mycobacterium tuberculosis* infection and the reasons thereof. Toward the end, we propose to have a set of guidelines for pursuing investigations to maintain uniformity in terms of early and late phase, MOI of infection, infection duration and incubation periods, the strain of mycobacteria, passage numbers, and so on, which all work as probable variables toward different readouts. Such a setup would, therefore, help in the smooth integration of information across laboratories toward a better understanding of the disease and possibilities of host-directed therapy.

## Introduction

Mitochondria are both the powerhouse of the cell and the hub of cell signaling pathways that maintain life and lead critical decisions that affect cell fate by their diverse physiological functions. Mitochondria are very dynamic structures and change from elongated oval to rounded circular bodies in a complex network. They are double membraned with the inner membrane displaying intricate invaginations, referred to as “cristae,” which harbor proteins involved in the relay of electrons in electron transport complexes (ETCs). Although the outer membrane is accessible to several metabolites, the inner membrane (IMM) is impermeable to metabolites by its lipid composition and forms a proton gradient to generate ATP.

The dynamic nature of the mitochondrial network is maintained by several homeostatic mechanisms such as fission, fusion, and mitophagy that vary in different cellular contexts such as the energy requirement of the cell and the pathological state. Several mitochondrial proteins of the outer membrane such as the mitochondrial fission and fusion proteins MFF, MFN1/2 (Daumke and Roux, 2017); BCL2 family proteins (Harris and Thompson, 2000), PINK/PARKIN (Mcwilliams and Muqit, 2017), ATG3/ATG12 (Radoshevich et al., 2010), ATF3 (Harris and Thompson, 2000; Matarrese et al., 2000; Radoshevich et al., 2010; Daumke and Roux, 2017; Bueno et al., 2018), Galectin-3 (Matarrese et al., 2000), α-Synuclein (Faustini et al., 2017), Cyclophilin-D (Javadov and Kuznetsov, 2013), inner membrane fusion protein OPA1 (Ehses et al., 2009), and other proteins, such as AMPK (Herzig and Shaw, 2018) control its homeostasis. AMPK is a well-known regulator of energy metabolism, and it also influences mitochondrial number by regulating mitochondrial fission factor (MFF). While the GTPase dependent MFN1 and MFN2 are outer mitochondrial membrane fusion proteins (Ishihara et al., 2004), OPA1 is the mitochondrial inner membrane fusion protein. The transcription factor NFκB controls mitochondrial dynamics through OPA1 in a non-canonical pathway (Laforge et al., 2016). The BCL2 family proteins maintain mitochondrial membrane potential and, therefore, stability. The damaged mitochondria are pinched off and recycled through mitophagy.

The intricate physical connections among various sub-cellular organelles and mitochondria with well-orchestrated cross-talk drive intracellular processes and communications (Daniele and Schiaffino, 2014). Each organelle has distinct membrane contact site (MCS)s through which they interact with each other to exchange molecules, such as Ca^2+^ (Daniele and Schiaffino, 2014), that regulate mitochondrial functions (Bravo-Sagua et al., 2017). A moderate increase in Ca^2+^ influx into mitochondria activates TCA cycle enzymes and promotes oxidative phosphorylation (OXPHOS). However, higher levels of Ca^2+^ trigger mitochondrial permeability transition pore (MPTP) and, thus, cell death. Also, Ca^2+^ levels impact mitochondrial mobility, wherein lower levels of Ca^2+^ cause free movement, and higher levels restrict their mobility, subsequently causing clustering. In the presence of higher levels of Ca^2+^, Ca^2+^-sensitive phosphatases dephosphorylate DRP1 leading to mitochondrial fission (Jahani-Asl and Slack, 2007).

The protein assembly in the double-layered membrane of mitochondria is complex with highly specialized functions and a highly regulated compartmentation. The outer membrane harbors several proteins that maintain membrane integrity and metabolite transporters. The inner membrane displays a more complex architecture with the presence of ETC proteins arranged as complexes (I–IV) within the invaginations of the inner membrane, cristae. The shape of the cristae changes in different metabolic and pathological states, resulting in altered bioenergetics. The structure of the inner membrane is maintained by proteins of the Mitochondrial Contact Site complex (MICOS). The outer membrane interacts with MICOS through SAM (Sorting and Assembly Machinery) complex (Reviewed in Cogliati et al., 2016). Loss of any component of MICOS causes altered shape and therefore altered OXPHOS. The displacement of these membrane proteins either by interaction with pathogen proteins or internal Ca^2+^ signaling leads to loss of MMP and release of inner mitochondrial membrane proteins. The released proteins initiate a chain of events leading to apoptosome formation and culminating in apoptosis or, in a worse scenario, the leakage of mito-ROS and subsequent necrosis.

**Figure 1:**
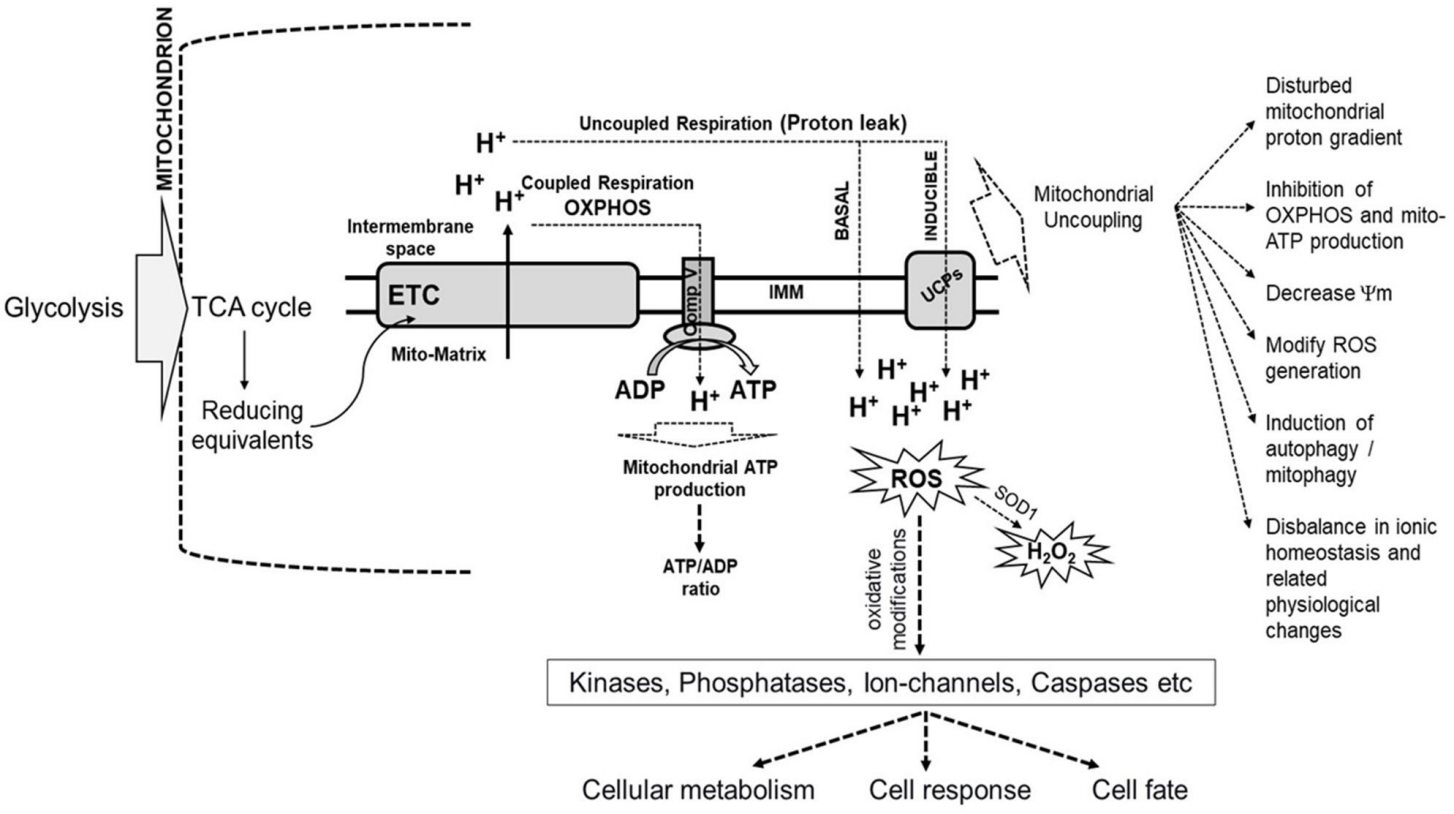
Schematic representation of the link between mitochondrial bioenergetics and cellular metabolism, cell response, and cell fate. The products of glycolysis from cytoplasm enter the TCA cycle in the mitochondrion, which generates reducing equivalents, such as NADH, the electrons from which enter the electron transport chain (ETC) located in the inner mitochondrial membrane (IMM). The bioenergetics of mitochondria is related to the coupled electron flow through ETC complexes with the generation of a proton gradient used by ATP synthase (Comp V) to produce ATP. Instead of getting transferred across, some electrons leak out through specific sites in ETC and interact with oxygen, leading to the generation of reactive oxygen species (ROS). Some protons routinely leak into the mitochondrial matrix across the inner mitochondrial membrane. This is called a proton leak, which can be basal or induced. Basal protein leak is attributed to the lipid bilayer of the inner mitochondrial membrane and the adenine nucleotide translocase, while induced proton leaks are mediated by uncoupling proteins (UCPs). Mild uncoupling and proton leak is protective to cells against generating excessive ROS. ROS causes oxidative modifications of important proteins such as kinases, phosphatases, ion-channels, caspases, amongst others, with the levels of ROS determining benefit or harm to the cell. The ROS produced by Complex III is released into the mitochondrial matrix and inter-membrane space (IMS). Superoxide dismutase (SOD1) can convert ROS present in IMS into H_2_O_2_, which can diffuse out of mitochondria and contribute to perturbations in cellular metabolism and pathological responses. Induction of mitochondrial uncoupling results in the dissipation of the mitochondrial proton gradient across IMM, and decrease mitochondrial membrane potential (Ψm), which modifies ROS generation, inhibits oxidative-phosphorylation (OXPHOS), induction of autophagy and many related changes in cellular physiology and cell fate (Demine et al., 2019).

The critical role of mitochondria as the cellular currency generator is facilitated through the TCA cycle by the generation of reducing equivalents, NADH, and FADH_2_ using the pyruvate that is transported from the cytosol into mitochondria through pyruvate carrier proteins. This highly regulated process drives the energy flux and can cause metabolic rewiring. The reducing equivalents, in turn, pass their labile electrons through the ETC arranged as complexes (I–IV) based on the redox potential of each carrier protein while releasing the energy, which is used to pump protons into the intermembrane space of mitochondria. The ultimate receiver of the electrons is an O_2_ molecule that forms superoxide radical, which immediately accepts protons to form two water molecules. The ATP synthase on the inner membrane uses the force in the proton gradient to fix the energy as ATP, referred to as aerobic respiration or oxidative phosphorylation (OXPHOS). Each NADH can generate 3 ATP, and FADH_2_ can generate 2 ATP. OXPHOS occurs during normal physiology and is highly regulated and sensitive to signaling events such as that mediated by Ca^2+^, which is essential for the activation of TCA cycle enzymes. Sometimes, electrons leak out through specific sites of ETC and react with oxygen, leading to the generation of ROS. Cellular ROS, primarily generated by mitochondria, is an essential physiological signaling molecule and a second messenger that arbitrate intracellular pathways (De Giusti et al., 2013; Brand, 2016). ROS causes oxidative modifications of critical proteins such as kinases, phosphatases, ion-channels, caspases, and the levels of ROS determine either benefit or harm to the cell. The ROS produced by Complex III is released into the mitochondrial matrix and inter-mitochondrial space (IMS). SOD1 can convert ROS present in IMS into H_2_O_2_, which can diffuse out of mitochondria and contribute to perturbations in cellular physiology and pathological responses. A small number of protons routinely leak into the mitochondrial matrix across the inner mitochondrial membrane, termed as proton leak, which can be basal or induced. The basal protein leak is attributed to the lipid bilayer of the inner mitochondrial membrane and the adenine nucleotide translocase (ANT), whereas induced proton leaks are mediated by uncoupling proteins (UCPs). Mild uncoupling and proton leak are protective to cells against generating excessive ROS. UCP-mediated proton leak protects the cell from oxidative stress due to the rapid production of ATP. The ROS generation and proton leak are closely associated, and therefore, the bioenergetics of mitochondria is intricately linked with cellular metabolism, response, and cell fate (Figure 1).

Several bacterial and viral infections often target mitochondria. Immunological responses are energy-consuming and tightly linked with energy metabolism. The understanding of immunometabolism is emerging as a paradigm shift in host-pathogen interaction in several pathological conditions such as cancer and diabetes. There are substantial data on host immunological, proteomic, transcriptional, and metabolic changes upon *Mtb* infection and many excellent recent reviews in the area of bioenergetics and immunometabolism of the host macrophages (Shi et al., 2016, 2019; Stutz et al., 2018; Russell et al., 2019; Ryan and O'neill, 2020). In this review, we have attempted to give a simplistic, yet comprehensive overview of the present-day understanding of the intricate correlations between *Mtb-*induced alterations in mitochondrial structural and functional dynamics together with rewiring of energy metabolism and bioenergetics that orients cell fate, immune response, and infection outcome along with a list of host-directed therapy targets involving mitochondrial functions. Understanding the link between mitochondrial bioenergetic changes and cell signaling pathways in greater detail would provide novel insights to devise host-directed therapies involving the restoration of mitochondrial function.

## *Mycobacterium tuberculosis* Infection Reallocates Mitochondrial Dynamics And Cellular Processes

*Mtb* infection of human macrophages is a complex and dynamic host-pathogen interaction with diverse outcomes based on the nature of the infecting strain and immune status of the host. In 90% of the cases, the bacteria are either cleared or enter dormancy. However, in 10% of the cases, the infection progresses by subversion of various host defenses, such as phagolysosome fusion and oxidative burst, as well as signaling pathways affecting cell fate as reviewed extensively (Divangahi et al., 2013; Srinivasan et al., 2014; Dubey, 2016; Mohareer et al., 2018). *Mtb* targets mitochondria, similar to other intracellular pathogens, as it is the hub of cellular signaling pathways and rewires its metabolism to suit its nutrient demands. During this process, *Mtb* alters the mitochondrial structure and function for its survival by targeting it with a battery of secreted factors. The alterations in mitochondria documented include either those as a part of host defense (early phase) or as a pathogenic manipulation (late phase) facilitating pathogen survival and dissemination. We discuss both these aspects, deliberating the role of mycobacterial factors wherever documented. It is essential to mention here that these studies have used a wide variety of *in-vitro, ex-vivo*, and *in-vivo* models, cell types, mycobacterial strains with different degrees of virulence, different infection burden (multiplicity of infection), and a range of post-infection observation time-points, making it challenging at times to correlate independent observations from different laboratories. Table 1 lists a few such examples for reference.

**Table 1:** List of diverse cell types used for investigation of host-pathogen interactions between *Mycobacterium tuberculosis* (and model systems thereof) and different cell types at the mitochondrial interface.

	**Cell type/model**	**Mycobacterial strain/ *Mtb* protein**	**MOI**	**Incubation post-infection**	**Impact on mitochondrial structure**	**Immunological consequence**	**Impact on non-mitochondrial bioenergetics**	**Impact on mitochondrial bioenergetics**	**Impact on cell fate**	**References**
Human	THP-1	*Mtb* H37Rv	10 5	24 h 24 h	Increased electron density, with a clear and vivid definition of the cristae	Modest increase in TNF-α	Increased as compared to uninfected cells	Mitochondrial membrane transition potential was significantly increased than H37Ra Increase in this ATP/ADP ratio augments activity of the mitochondrial electron transport chain Decreased ATP (mitochondrial respiration) Oxygen consumption rate as in basal respiration, total respiration, proton leak, and TCA are all reduced as compared to uninfected THP-1	Modest increase in both apoptosis and necrosis	(Riendeau and Kornfeld, 2003; Jamwal et al., 2013; Asalla et al., 2017; Cumming et al., 2018)
		Dead *Mtb*	5	24 h			Slightly reduced as compared to untreated cells	Oxygen consumption rate as in basal respiration, total respiration, proton leak, and TCA are reduced slightly as compared to uninfected THP-1 but much higher than *Mtb* or BCG	No effect	(Cumming et al., 2018)
		*Mtb* H37Ra	10	24 h	A significant reduction in the electron density of the matrix outlines of the cristae were distinct and there was no gross pathology of the mitochondria	TNF-α		Mitochondrial membrane potential was significantly lower than H37Rv Decrease in this ATP/ADP ratio as compared to uninfected cells Decreased mitochondrial electron transport chain	Apoptosis	(Jamwal et al., 2013)
		BCG	10 5 1	24 h 2-72 h		Significant increase in TNF-α Significant release of CCL2 Activates macrophages	Non-mitochondrial respiration is more than that induced by *Mtb*	Generates superoxide radicals Oxygen consumption rate as in basal respiration, total respiration, proton leak, and TCA are all reduced as compared to uninfected THP-1but higher than *Mtb*	Significant increase in apoptosis	(Riendeau and Kornfeld, 2003; Méndez-Samperio et al., 2010; Chávez-Galán et al., 2016; Asalla et al., 2017; Cumming et al., 2018)
	U937	*Mtb* H37Rv *Mtb* H37Ra	10 10	1–5 days 1–5 days				ΔMMP increased greater with H37Rv than H37Ra but similarly with MOI > 5	Modest increase in apoptosis and necrosis (day3 onwards) Significant increase in apoptosis and not necrosis (day3 onwards)	(Danelishvili et al., 2003)
	Jurkat T cells	rRv1818c-Tat	1–15 μg/ml	24 h	Rv1818c localizes to mitochondria				Caspase dependent apoptosis	(Balaji et al., 2007)
	RD (sarcoma)	Transient expression of PE_PGRS33	1 μg plasmid with Lipofectamine	24 h	PE_PGRS33 localizes to mitochondria				Apoptosis (24 h onwards) and necrosis (late stage-48 h onwards)	(Cadieux et al., 2011)
	PBMC derived macrophages	*Mtb* H37Rv *Mtb* H37Ra LpqH	2–10 2–10 0.5–50 μg/ml	0–48 h 0–48 h 24 h				At MOI = 5, H37Rv caused more MMP and cytC release at 6 h At MOI-10, both H37Rv and H37Ra caused an equal release of cytC and back at 48 h	Modest increase in apoptosis 48 h and necrosis(72 h) Significant increase in apoptosis and modest increase in necrosis Caspase dependent apoptosis	(Chen et al., 2006; Sánchez et al., 2012)
	PBMC derived dendritic cells	*Mtb* H37Rv *Mtb* H37Ra Dead *Mtb*	1–10 1–10 1–10	24–48 h 24–48 h 24–48 h		DC maturation Il-1, Il-6, TNF-α. DC maturation Il-1, Il-6, TNF- α No DC maturation IL-6, IL-8, TNF- α secreted		Non-apoptotic cell death Non-apoptotic cell death No cell death	Caspase independent	(Ryan et al., 2011)
	alveolar epithelial cells (A549 type II epithelial cells)	*Mtb* Erdman *Mtb* CDC1551 *M. bovis-BCG* *Mtb* Erdman, an ESAT-6 deletion mutant *M. smegmatis*	10, 100 10 10, 100 50	6–24 h 48 h 6–48 h 6–24 h 48 h	No effect Fragmented, appear as spherical structures Decrease in mitochondrial mass (pronounced effect with MOI = 100) No effect on mitochondrial structure and mass No effect	No inflammasome activation		No effect No effect Significant decrease in MMP	Necrosis (post 48 h) Necrosis Necrosis (post 5 days) No cytotoxicity up to MOI = 50	(Dobos et al., 2000; Fine-Coulson et al., 2015)
Murine	macrophage cell line RAW 264.7	H37Rv rRv3261 rHBHA BCG	10–20 10 μg/ml 10	1–4 days 24 h 24 h	Localizes to mitochondria	Dose dependent LDH release ROS production TNF, IL-6, MCP-1 TNF-α and IL-6 are secreted		Loss of membrane potential, Bax translocation, Cyt C release Decrease in MMP, bax translocation, Cyt C release Decrease in MMP	Necrosis in a dose and time-dependent manner Caspase-3/9 dependent apoptosis Caspase dependent apoptosis Caspase dependent apoptosis	(Sohn et al., 2011; Wu et al., 2014; Jee et al., 2017; Lee et al., 2020)

*The above list includes a diverse array of cell types used in mycobacterial research with details of variables of experimental conditions (Specific strain, MOI, incubation time post-infection) with mitochondria-associated readouts such as morphological changes, Bioenergetic changes, and cell fate. It is be noted that it is not an exhaustive list of all model cell types and mycobacterial strains used in mycobacterial research*.

### Alterations in Morphology and Cellular Distribution of Mitochondria

Mitochondrial effector functions are coupled to its altered morphology and distribution dynamics, which are regulated by fusion and fission events (Escobar-Henriques et al., 2006). Healthy mitochondria are long rod-shaped with an interconnected network, whereas affected mitochondria are either spherical or punctuated (Cataldo et al., 2010). Several researchers have observed changes in mitochondrial structure upon mycobacterial infection of human and mouse macrophages, both *in vitro* and *in vivo*, wherein their structure altered from rod shape to spherical or ovoid (Abarca-Rojano et al., 2003; Jamwal et al., 2013; Asalla et al., 2017). *Mtb* induced fragmented mitochondrial morphology along with increased levels of mitochondrial fission proteins, DRP1, and p-MFF, but decreased levels of the fusion protein, MFN2 in a dose-dependent manner during *Mtb* infection of primary mouse cells (BMDM) and macrophage cell line, RAW 264.7. siRNA mediated knockdown of MFN2 suppressed intracellular *Mtb* survival, wherein, it was proposed that by disrupting the mitochondrial network, MFN2 induces apoptosis of the host cell (Lee et al., 2019). Also, defects in mitochondrial fusion are associated with reduced respiration and loss of mitochondrial membrane potential (MMP/ΔΨ) in human peripheral blood mononuclear cells (PBMC) (Chen et al., 2005). Mitochondria tend to get aggregated around the *Mtb* laden phagosomes and form an elongated structure in an infected macrophage, although the physiological significance of this association is not clear. Additionally, the virulent nature of the infecting mycobacterial strain also influenced the bearing on mitochondrial morphology (Jamwal et al., 2013). For instance, mitochondria of THP-1 cells infected with virulent *Mtb-*H37Rv strain showed higher electron density with clear cristae in contrast to those of THP-1 cells infected with avirulent *Mtb*-H37Ra, wherein mitochondria showed decreased electron density, with distinguishable outlines of the cristae (Jamwal et al., 2013). Similarly, changes in mitochondrial morphology were observed in lung epithelial cells infected with *Mtb* but not with the avirulent TB vaccine candidate, *M. bovis-*BCG. ESAT-6, one of the significant virulent factors of *Mtb*, absent in *M. bovis-*BCG, was proposed to be associated with the altered mitochondrial morphology (Fine-Coulson et al., 2015). Restoration of either fusion-fission genes (Lee et al., 2019) or restoration of the mitochondrial parameters to a steady state by small-molecule M1 reinstated the ability of macrophages to clear intracellular bacillary load (Asalla et al., 2017), demonstrating the significance of mitochondrial morphological changes in regulating mycobacterial survival inside macrophages.

### Changes in the Mitochondrial Proteome

To correlate the phenotypic and functional alterations with mitochondrial protein composition during *Mtb* infection, comparative proteomic studies were undertaken in PMA differentiated THP-1 cells infected either with virulent H37Rv or avirulent strains of *Mtb* after 24 h, which, as expected, differed with the virulent/avirulent nature of mycobacterial strain used. Infection with the virulent H37Rv strain showed alteration in the levels of various components of ETC, such as mitochondrial FoF1 ATP synthase subunits (ATP50), NADH dehydrogenase [ubiquinone] iron-sulfur protein, Succinate dehydrogenase [ubiquinone] flavoprotein subunit, Cytochrome b-c1 complex subunits, Cytochrome C Oxidase (COX) subunits, and NADH-Cytochrome b5 reductase. The dataset provided in the report by Jamwal et al. (2013), lists several proteins that are known to localize to various sub-fractions of mitochondria, offering cues to changes in outer membrane composition, where levels of several transporter proteins, enzymes, and integral membrane or structural proteins were altered upon *Mtb* (H37Rv or H37Ra) infections. Such proteins included (i) channel or transporter proteins, such as Voltage-dependent anion-selective channel protein, Mitochondrial import receptor subunit TOM22, Metaxin-1, Chloride intracellular channel protein-4, Non-specific lipid-transfer protein, Carnitine O-palmitoyltransferase-1, Translocator protein, or (ii) enzymes, such as long-chain-fatty-acid–CoA ligase, Serine/threonine-protein phosphatase PGAM5, and (iii) other proteins, to name a few, Regulator of microtubule dynamics protein-3, Ras-related protein Rab-32, Apoptosis-inducing factor-2, Sorting and assembly machinery component SAM50, Endophilin-B1, Synaptic vesicle membrane protein VAT-1, and others. It will be interesting to dissect the independent role of these proteins in *Mtb* pathogenesis and in regulating bioenergetic changes during infection. SAM50 which is instrumental in the structural maintenance of mitochondrial cristae and correct assembly of the mitochondrial respiratory chain complexes (Ott et al., 2012, 2015) or Endophilin-B1 that is vital for the conservation of mitochondrial morphology (Karbowski et al., 2004).

Besides perturbations in the proteins of ETC, there are differences in the inner membrane and mitochondrial matrix proteins exemplified by hydroxy acyl-CoA dehydrogenase (HADHA), ATPase family AAA domain-containing protein, Sulfide: quinone oxidoreductase (SQRDL), Proto-oncogene tyrosine-protein kinase Src, Acylglycerol kinase, DLD (dihydrolipoamide dehydrogenase), Acetyl-CoA acetyltransferase (ACAT1), redox maintaining proteins of the mitochondrial matrix such as Peroxiredoxin-1 (PRDX1), Thioredoxin-dependent peroxide reductase (PRDX3) and others (Ragno et al., 1998; Jamwal et al., 2013). Comparative proteomic changes induced in host mitochondrial proteome by avirulent *Mtb* H37Ra vs. virulent *Mtb* H37Rv were analyzed by Jamwal et al., wherein a few mitochondrial proteins showed opposite regulation, including anti-apoptotic ATAD3A that was down-regulated in H37Ra infected THP-1 cells but upregulated in H37Rv, consistent with their ability to induce or suppress apoptosis. Also, the proteins of NO detoxification, such as SQRDL, shows a similar trend, wherein its levels are low in H37Rv infected cells, thereby promoting H_2_S and mycobacterial persistence (discussed in a later section) and higher in H37Ra infected cells resulting in oxidation of H_2_S and consequent antimicrobial activity resulting in clearance. Another protein that is regulated inversely is HADHA that is involved in the beta-oxidation of fatty acids. Their low levels in H37Rv infected cells and high levels in H37Ra explains the decreased fatty acid catabolism in H37Rv infected cells than H37Ra and consequent lipid droplet accumulation in H37Rv and not H37Ra (Singh et al., 2012). The down-regulated catabolism vis-a-vis HADHA is further complemented by increased ASCL1 that catalyzes the conversion of long-chain fatty acids to fatty acyl-CoAs and further lipid droplet formation.

### Changes in Mitochondrial Bioenergetics

With the above account, it will not be unseemly to presume that the alterations in the mitochondrial proteome may affect cristae maintenance and mitochondrial membrane composition, influencing membrane permeability that may lead to differences in the transmembrane proton gradient affecting transmembrane electric potential, pH gradient, and resultant ATP generation. This may also result in the release (or leak) of mitochondrial proteins into the cytoplasm or entry of cytoplasmic factors into mitochondria, perturbing mitochondrial physiology and bioenergetics. Infection of human monocyte-derived macrophages with the pathogenic strain of *Mtb*, H37Rv, caused mitochondrial inner membrane damage resulting in mitochondrial permeability transition (MPT) leading to necrosis, while the non-pathogenic mycobacteria majorly induced apoptosis which was not associated with MPT changes (Chen et al., 2006). Taken together with earlier observations, upon infection with either virulent or avirulent *Mtb* similar mitochondrial changes were observed in the early stages. In contrast, differential changes were observed in the late stages, suggesting that the early changes in mitochondria are host driven, whereas the pathogen induces the later changes during successful infection.

With the availability of real-time live-cell metabolic assay platforms for measuring oxygen consumption rate (OCR) and extracellular acidification rate (ECAR) for examining mitochondrial respiration and glycolysis, respectively, mitochondrial bioenergetics have been investigated in detail. Perturbation in bioenergetics of mitochondria is largely a measure of some direct and derived parameters briefly discussed below and elaborated in the reference (Mills et al., 2016; Cumming et al., 2018). The assays rely on the usage of specific inhibitors such as oligomycin (ATP synthase inhibitor), rotenone (complex I inhibitor), and antimycin A (complex III inhibitor) and uncouplers like carbonyl cyanide-4-(trifluoromethoxy) phenylhydrazone (FCCP) that uncouples ATP synthesis from electron transport. The usage of these chemicals helps in distinguishing mitochondrial-dependent and non-mitochondrial respiration and energy metabolism. Some of the basic parameters that have been compared between uninfected/unstimulated vs. infected/stimulated macrophages include non-mitochondrial respiration (OCR after inhibiting complex I and III), ATP-linked respiration (OCR after inhibiting ATP synthase), proton leak (ATP-linked OCR minus NMR); maximal respiration (OCR after uncoupling ATP synthesis from ETC) and spare respiratory capacity (the difference between maximal respiration and basal respiration). The studies applied these well-established protocols to measure the perturbations in host mitochondrial bioenergetics systematically suggesting bioenergetic links to macrophage pro-inflammatory response and mycobacterial infections (Mills et al., 2016; Cumming et al., 2018). Glycolytic parameters were measured as a function of ECAR with non-glycolytic acidification as the measurement of ECAR before adding glucose, and maximal glycolytic capacity (ECAR after inhibiting ATP synthase minus non-glycolytic acidification). While all observations in these studies can be summed up as rewiring of host energy metabolism upon mycobacterial infection, the degree of such changes varied with the cell type used, the pathogenicity of mycobacteria, multiplicity of infection (MoI), time point of analysis, and treatment with live or dead bacteria (Table 1).

Studying the correlation of bioenergetics with immune response, Mills et al. used LPS stimulated murine BMDMs and found that a mitochondrial metabolic process of oxidation of succinate by succinate dehydrogenase (SDH) affected the expression of pro- or anti-inflammatory cytokines. They linked that LPS stimulation shifted OXPHOS mediated ATP generation to glycolysis, increased MMP, elevated mitoROS production, and increased succinate oxidation *via* succinate dehydrogenase (SDH) that regulated the expression of pro- and anti-inflammatory genes. It was observed that elevated ATP production *via* glycolysis helped to augment MMP that was instrumental for LPS mediated IL-1β expression (Mills et al., 2016). The relationship between host energy metabolism and immunological consequences to mycobacterial infections has been discussed in a separate section. Like LPS treatment, some studies suggested that treatment with live or killed mycobacteria also caused a metabolic switch from OXPHOS to aerobic glycolysis, a Warburg-like phenomenon (Appelberg et al., 2015; Lachmandas et al., 2016b). Cummings et al. performed a real-time study on a non-invasive bioenergetic platform for investigating mitochondrial respiration and glycolysis during mycobacterial infection of macrophages. They used two cell types (PMA differentiated THP-1 and hMDMs) and three types of treatments [with live virulent *Mtb*, the non-virulent vaccine strain, *M. bovis* BCG (BCG), and dead-*Mtb* preparation]. These observations showed that treated macrophages have disparate respiratory differences, which depended on cell type, the burden of infection, and pathogenicity of mycobacteria (Cumming et al., 2018). Like others, they also observed that *Mtb* infection depressed the rate of OXPHOS in macrophages. Spare respiratory capacity (SRC), which is important for ATP production in situations when the energy demand exceeds supply (such as when a cell has added workload or is under stress), was decreased by the pathogenic *Mtb*, while the vaccine strain *M. bovis*-BCG increased the same in hMDMs. However, non-mitochondrial respiration (NMR) was amplified in hMDMs by either *Mtb* or *M. bovis*-BCG infections. Increased NMR suggested a shift from OXPHOS to glycolysis. The measurement of glycolytic parameters, interestingly, did not support the earlier observation by several labs that *Mtb* infection promoted aerobic glycolysis. In contrast to that, this study showed that virulent *Mtb* dramatically decreased glycolytic parameters, such as glycolytic proton efflux rates of both THP-1 and hMDMs.

However, *M. bovis*-BCG and dead *Mtb* infection of hMDMs increased glucose metabolism acidification, glycolytic capacity, basal, and compensatory glycolytic rates. The overall ATP generation was reduced during *Mtb* infection. The bioenergetic phenograms (basal OCR as a function of ECAR) suggested that all treatments drive macrophages toward quiescent phenotype, but is most pronounced for live virulent *Mtb* as compared to *M. bovis*-BCG infection or dead-*Mtb* treatment. In contrast, the ATP/ADP ratios reported by Jamwal et al. under similar conditions reveal a reverse trend, wherein the virulent *Mtb* showed a significant increase in ATP/ADP ratio as compared to avirulent *Mtb* H37Ra (Jamwal et al., 2013). LC-MS/MS, in conjunction with ^13^C-tracing, showed reduced enrichment of TCA metabolites in the *Mtb*-infected hMDMs suggesting a decelerated bioenergetic metabolism (Cumming et al., 2018). While this study established robust quantifiable bioenergetic parameters, some of the observations also challenged the general dogma in the field. As the experiments are very sensitive to a variety of parameters, one requires to investigate if the conflicting observations are due to different experimental conditions.

### Alterations in the Host Mitochondrial Signaling and Cell Fate by Mycobacterial Proteins

**Figure 2:**
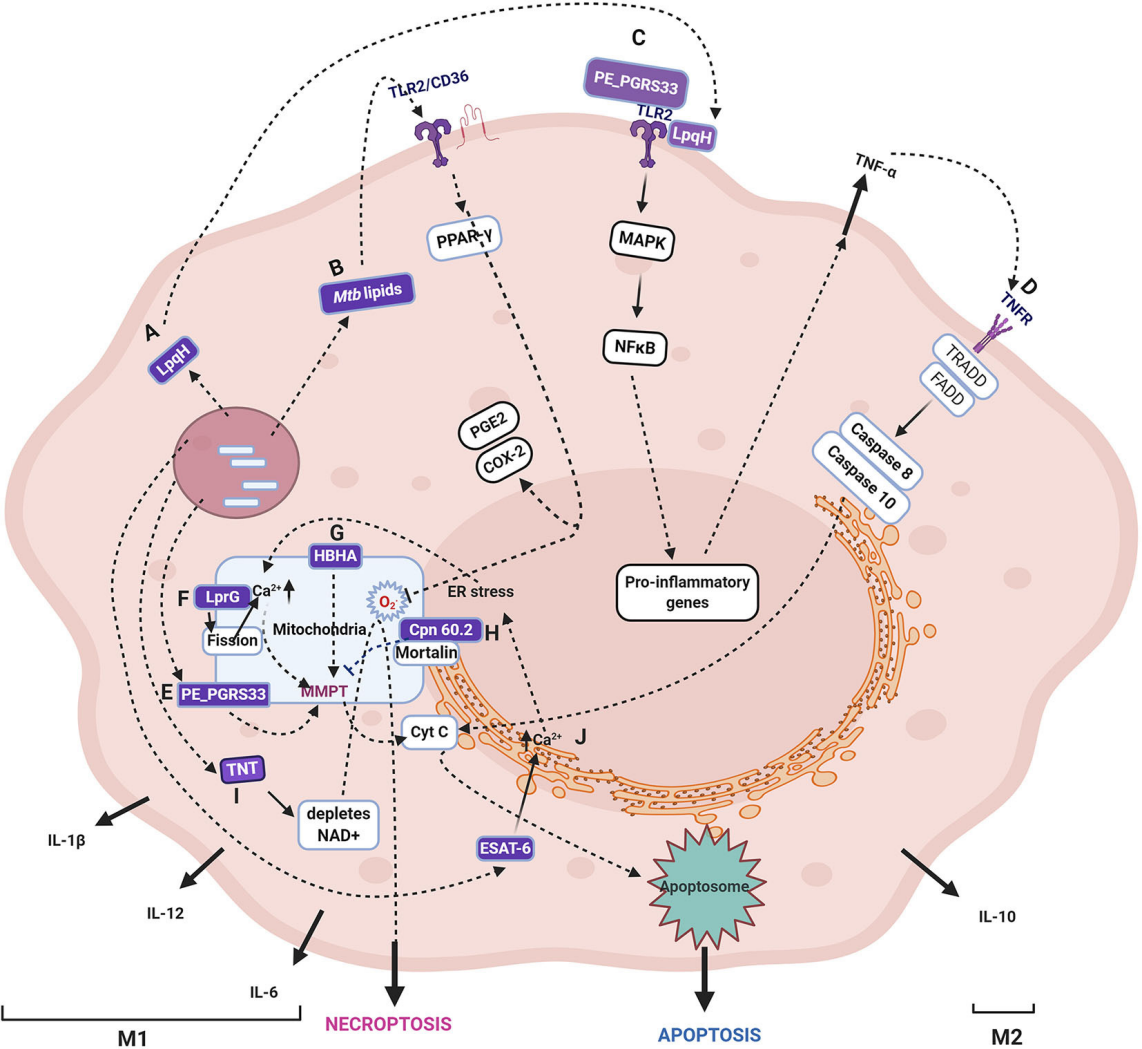
Schematic representation of various cell fates upon *Mycobacterium tuberculosis* infection. The figure depicts the *Mtb* proteins that are demonstrated to translocate to mitochondria and their role in induction or inhibition of cell death modalities. The proteins, lipids, and carbohydrates that are either secreted or on the surface of *Mtb* are recognized by host pattern recognition receptors (majorly TLR2). The several proteins secreted by *Mtb* target various host organelles, of which mitochondria is a crucial target. Specific *Mtb* proteins, as discussed in the text, are reported to translocate to the mitochondrial membrane, wherein they cause a change in MMP, leading to Cytochrome C release and, subsequently, apoptosis. The specific pathway differs for each *Mtb* protein with differences in the source of perturbation, such as the generation of ROS, changes in Ca^2+^ dynamics, and mitochondrial fission. Some proteins are not reported to translocate to mitochondria, but their activities ripple their effect on mitochondria, such as TNT, which causes NAD+ depletion leading to increased ROS generation in mitochondria. The receptors, including TLR, TNF, IL-1R, NOD, signal through various combinations of adaptor proteins that result in specific signaling pathways and diverse outcomes such as apoptosis, necrosis, necroptosis, and autophagy as described in the text. The figure depicts the *Mtb* proteins that are demonstrated to translocate to mitochondria and their role in the induction or inhibition of cell death modalities. One of the several *Mtb* proteins that inhibit apoptosis by interacting with mitochondrial mortalin is Cpn60.2. **(H)**. LpqH **(A)** and PE_PGRS33 **(D)** interact with TLR2 **(C)** and induces apoptosis through TNF-α. In the intracellular milieu, *Mtb* factors upon translocation to mitochondria and causes a change in MMP and leads to apoptosis **(E)**. *Mtb* lipids such as Man-LAM can bind to TLR2/CD36 and signal PPAR- γ resulting in proinflammatory cytokines **(B)**. HBHA is another secreted *Mtb* protein that causes apoptosis by changing MMP **(G)**. LprG causes changes in Ca^2+^ dynamics and mitochondrial fission that leads to MMPT and, thus, apoptosis **(F)**. One of the secreted proteins, TNT, hydrolyzes NAD resulting in the accumulation of ROS, and finally, necroptosis **(I)**. The major secreted virulent protein, ESAT-6, causes ER stress that is transferred to mitochondria and finally culminating in apoptosis **(J)**. It may be noted that the early phase of infection is dominated by either apoptosis that leads to bacterial clearance or anti-apoptosis that promotes bacterial survival and growth (M1). Once *Mtb* survives the host pro-inflammatory responses, it takes over by modulating the host metabolism by anti-inflammatory cytokines and alterations in lipid metabolism that inhibit apoptosis and autophagy coinciding with the M2 phase. As cell death is one of the major outcomes of perturbation in mitochondrial activities with or without getting targeted by mycobacterial factors, for a comprehensive list of cell death modulators from *Mtb*, please refer (Mohareer et al., 2018). The mycobacterial proteins are marked in purple boxes. The figure has been made using the Biorender app.

*Mtb* modulates several host innate immune signaling pathways by secretion of numerous proteins (Figure 2), which gain access to the host cytosol by the phagosomal pores created by the concerted activity of *Mtb* ESAT-6 and PDIM (Augenstreich et al., 2020). These mycobacterial proteins subsequently target various sub-cellular organelles such as ER, mitochondria, nucleus, and orchestrate an anti-apoptotic state of the host during early infection (Bussi and Gutierrez, 2019). One such representative *Mtb* protein that targets host mitochondria and inhibits cell death is Cpn60.2 (GroEL2), a cytosolic chaperone protein. Cpn60.2 is released from the bacterial surface that crosses the phagolysosome membrane and enters the host cytosol and further reaches mitochondria where it interacts with the mitochondrial mortalin protein, a member of the HSP-70 family, involved in apoptosis. Cpn60.2 blocks apoptosis induced by mortalin and thus plays an anti-apoptotic role and promotes bacterial persistence inside the host (Joseph et al., 2017) (Path H, Figure 2).

During the early host-pathogen interaction, several cell death modalities, most notedly, apoptosis, necrosis, necroptosis, pyroptosis, ferroptosis as well as autophagy, are induced either by host or *Mtb* that decide the outcome of infection and in case of a successful establishment, *Mtb* persists and disseminates. Upon infection of macrophages by either virulent or avirulent strains of mycobacteria, apoptosis is induced by the host wherein the MMP is altered, and Cytochrome-C is released. It has been consistently observed that avirulent species are better apoptosis inducers than *Mtb*-H37Rv in primary cells and cell lines of both human and mouse as well as bone marrow-derived macrophages (Keane et al., 2000) suggesting that virulent *Mtb* also inhibits apoptosis (reviewed in Briken and Miller, 2008). Further, only virulent *Mtb* can damage the mitochondrial inner membrane during late-phase, leading to necrosis (Chen et al., 2006).

The role of mitochondria in the progression of various cell death pathways identified to date has long been established and reviewed by several authors (Parandhaman and Narayanan, 2014; Mohareer et al., 2018). Presently, we focus on the changes in mitochondria induced by *Mtb*, such as increased ROS, Ca^2+^ dynamics, loss of MMP and, fission events, toward the various cell fate outcomes with particular emphasis on the role of *Mtb* factors during the induction of the cell death pathways as well as autophagy.

#### ROS as a Signaling Molecule

One of the very well-studied factors for host inducted apoptosis is ROS, which is also closely associated with mitochondrial bioenergetics. It is generated as an innate immune response to an intracellular infection of activated macrophages. NADP oxidase (subunits p67 phox and p47 phox) that is upregulated upon *Mtb* infection is recruited to phagosomes and mediates respiratory burst (Shui et al., 2009).

Additionally, the activated macrophage reprograms its metabolism, during which there are specific breakpoints, discussed in a later section, which leads to the accumulation of mito-ROS. The ROS are also channeled to phagosomes from the surrounding mitochondrial cluster, and thus the resident *Mtb* is exposed to ROS (Jamwal et al., 2013). The excess ROS, which is not quenched by *Mtb* factors, signal toward extrinsic apoptosis pathway dependent on TNF-α (Miller et al., 2010) (Figure 2, path D). The mito-ROS further leads to MMPT and release of Cytochrome C and therefore activates the intrinsic apoptosis pathway. One *Mtb* factor that is known to induce mito-ROS is *Mtb*-TNT (Figure 2, path I). MAV2054, an *M. avium* protein, is also reported to induce ROS-mediated caspase-dependent apoptosis through JNK activation as well as the loss of MMP and release of Cytochrome C. Besides, IL-6, TNF-α, and monocyte chemoattractant protein-1 (MCP-1) were also induced by MAV2054 (Lee et al., 2016). The extent of ROS defines the cell fate ranging from quenching by anti-oxidant enzymes and proteins to apoptosis, apoptosis variants (pyroptosis, necroptosis), and necrosis. Other *Mtb* proteins known to induce ROS include the PE25/PPE41 complex that induces necrosis preceding dissemination (Tundup et al., 2014), ESAT-6, and ZMP-1 (at higher bacterial burden) also induce necrosis.

#### Calcium Ions as Signaling Molecules

The other signaling factor toward cell fate decision is the second messenger Ca^2+^. Certain *Mtb* factors, such as ESAT-6, cause ER stress resulting in disruption of Ca^2+^ homeostasis (Choi et al., 2010) that spirals into a disturbance in Ca^2+^ homeostasis of mitochondria. Increased Ca^2+^ inside the mitochondrial matrix causes loss of MMP and further leakage of mitochondrial contents and, thus, apoptosis (Path J, Figure 2). ER stress also stimulates M1 polarization of macrophages, discussed in later sections, and results in the secretion of various cytokines such as TNF-α, IL-1, IL-6, IL-12, and IL-23. Among these, TNF-α plays a significant role in the signaling of apoptosis through the extrinsic pathway (reviewed in Lam et al., 2017). One of the first *Mtb* proteins that were demonstrated to co-localize with mitochondria, PE_PGRS33, causes cell death in the host macrophages (Basu et al., 2007; Cadieux et al., 2011) (Path E, Figure 2). PE_PGRS33 interacts with TLR2, and the signaling culminates in the secretion of TNF-α and consequently apoptosis (Path C, Figure 2). *Mtb* Rv1411c (LprG/p27) induces mitochondrial fission and changes mitochondrial Ca^2+^ dynamics and lowers the host rate of respiration (Path F, Figure 2). On the contrary, Rv1818c (PE_PGRS33) promotes mitochondrial fusion with no effect on Ca^2+^ uptake and respiratory rate of the cell (Aguilar-López et al., 2019). Similar effects were observed upon infection with *M. smegmatis* overexpressing MAV2054 in bone marrow-derived macrophages. MAV2054 limits the growth of *M. smegmatis* inside the mice and promotes the survival rate of mice (Lee et al., 2016). LpqH, an *Mtb* 19 kDa lipoprotein, activates the TLR2 receptor leading to the activation of caspase-8 and caspase-3 (Path C, Figure 2). Along with the caspase-dependent pathway, LpqH also induces a caspase-independent pathway in the macrophage apoptosis process (Sánchez et al., 2012). Ca^2+^ dynamics and MMP are closely linked and interdependent.

#### Decrease in Mitochondrial Membrane Potential as a Signal

The loss of mitochondrial membrane integrity is crucial for the progression and persistence of infection (Duan et al., 2002), which was evident in the infection of both virulent (H37Rv) and avirulent (H37Ra) *Mtb*. Only virulent *Mtb* that progress to successful infection can cause mitochondrial inner membrane damage (Chen et al., 2006). Also, the extent of MMPT defines the mode of cell death with lower differences causing apoptosis and higher MMPT, causing necrosis, as defined by the bacterial load in the cell. Several mitochondrial outer membrane proteins that maintain mitochondrial membrane integrity, such as BCL2 family proteins, are upregulated, and those that destabilize MMP are down-regulated. For instance, BCL2 is upregulated, and Bax is downregulated (Mogga et al., 2002). Heparin-binding heme agglutinin protein (HBHA) is a secreted as well as a surface-associated protein of mycobacteria that is transported to mitochondria during *Mtb* infection. HBHA treatment causes loss of MMP and release of cytochrome C, leading to apoptosis with characteristic patterns (Sohn et al., 2011) (Path G, Figure 2). Pathogenic mycobacteria can also avert host induced apoptosis and drive the host toward necrosis but not non-pathogenic mycobacteria (Fratazzi et al., 1997; Pais and Appelberg, 2000). Most of the mitochondria targetting *Mtb* proteins are reported to induce cell death, especially toward necrosis except Cpn60.2 that inhibits apoptosis.

In recent times, the understanding of cell death mechanisms is continuously evolving as variants that differ in classical apoptosis, and necrosis mechanisms such as pyroptosis, necroptosis, ferroptosis pyronecrosis, and others were observed. These patterns arise due to a multitude of stimuli with cross-connected pathways. During *Mtb* infection, several researchers have identified several such variations. A caspase-independent mode of cell death was reported in macrophages upon *Mtb* infection that cleaved Bid and could be blocked by serine protease inhibitors (O'sullivan et al., 2007). *Mtb* specific factors induce necroptosis through RIPK3 and BCL-XL by blocking apoptosis and caspase-8 (Zhao et al., 2017). Also, the depletion of NAD+, induced by *Mtb*-TNT (NAD glycohydrolase), triggers a necroptosis pathway by enhanced ROS generation (Pajuelo et al., 2020) (Path I, Figure 2). One such secreted *Mtb* factor ZMP-1, a metalloprotease that has been demonstrated to cause dissemination in the Zebrafish model (Mani et al., 2016). Besides these factors, several potential mycobacterial factors have been predicted to target mitochondria (Rana et al., 2015). Although *Mtb* induced perturbations of mitochondrial OXPHOS and ATP generation are known, the role of specific *Mtb* proteins, such as oxidoreductases and thioredoxins, is yet to be investigated.

Successful progression of *Mtb* infection occurs by inhibition of apoptosis. *Mtb* infected cells can inhibit apoptosis induced by an external agent, for instance, TNF-α. The robust anti-apoptotic mechanism can be co-related to altered/dysfunctional mitochondria, which fail to carry out apoptotic signaling. Following persistence, *Mtb* replicates in the macrophage and induces necrosis rather than apoptosis that is essential for bacterial dissemination. Host cell apoptosis has contradictory observations on bacterial killing and disease outcome. While host induced apoptosis is thought to clear the infection, the bacteria are alive in the apoptotic bodies that are ultimately killed by highly robust hydrolytic enzymes upon efferocytosis (Nagata, 2018). Apoptosis also helps in antigen cross-presentation to T cells and helps in immune activation. Both of these processes work as pro-host strategies. On the contrary, apoptosis induced by *Mtb* helps the pathogen in escaping from the granuloma for disseminating from the host. While in the granuloma, *Mtb* secretes ESAT-6 that induces expression of MMP (matrix metalloprotease) on epithelial cells, and thereby the *Mtb* masked in apoptotic bodies escape the highly contained granuloma. Recombinant variants of *M. bovis*-BCG that express apoptosis inducers have been demonstrated to have better efficacy than *M. bovis*-BCG alone as the antigen presentation, and immune activation is enhanced by increasing apoptosis of infected cells (Gengenbacher et al., 2016). The overall importance of apoptosis as a pro-host is clear from the studies with IPR-1 (Intracellular Pathogen Resistance gene), knockout mouse (a gene that is crucial for apoptosis) which are highly susceptible to *Mtb* infection. Also, Alox-5 (5-lipoxygenase) knockout mouse, which is defective of necrosis, is resistant to *Mtb* infection (Bafica et al., 2005). Taken together, the induction of apoptosis in the early phase inside the alveolar macrophages helps in the clearance of bacteria and a pro-host process, but whereas inside the granuloma works as a pro-pathogen process. Investigation of the temporal regulation of inducers of apoptosis would provide better insights into the process.

In addition to cell death, mitochondrial outer membrane proteins interact and orchestrate during induction of autophagy (reviewed in detail in Mohareer et al., 2018) in response to mycobacterial infection leading to its clearance (Khan and Jagannath, 2017). Mitochondria undergo autophagy to maintain homeostasis wherein defective mitochondria are recycled. As mitochondria become damaged in consequence of mycobacterial infection, autophagy is activated that not only recycles mitochondria but also clears the mycobacterial infection. PINK1 and PARKIN proteins play a significant role in the regulation of autophagy. PINK1 accumulates in the damaged mitochondrial outer membrane and phosphorylates PARKIN that leads to activation of ubiquitin ligase and polyubiquitinates mitochondrial outer membrane proteins for its degradation (Narendra and Youle, 2011; Roca-Agujetas et al., 2019). Deletion of the *parkin* gene leads to severe susceptibility to intracellular pathogens, demonstrating the role of PARKIN in innate immunity (Manzanillo et al., 2013). However, successful *Mtb* infection evades autophagic clearance by a battery of secreted proteins (reviewed in detail in Mohareer et al., 2018).

### Rewiring of Host Energy Metabolism and Immunological Consequences

The correlation of mitochondrial bioenergetics with immune response was evident with the study by Mills et al. where the secretion of pro-inflammatory cytokine IL-1β in LPS treated murine BMDMs was allied to shift of OXPHOS mediated ATP generation to glycolysis, increased mitochondrial membrane potential, elevated mitoROS production, and increased succinate oxidation via succinate dehydrogenase (SDH) (Mantovani et al., 2004; Lachmandas et al., 2016a,b; Shi et al., 2019).

#### M1 Phase: The Host Defense Phase

**Figure 3:**
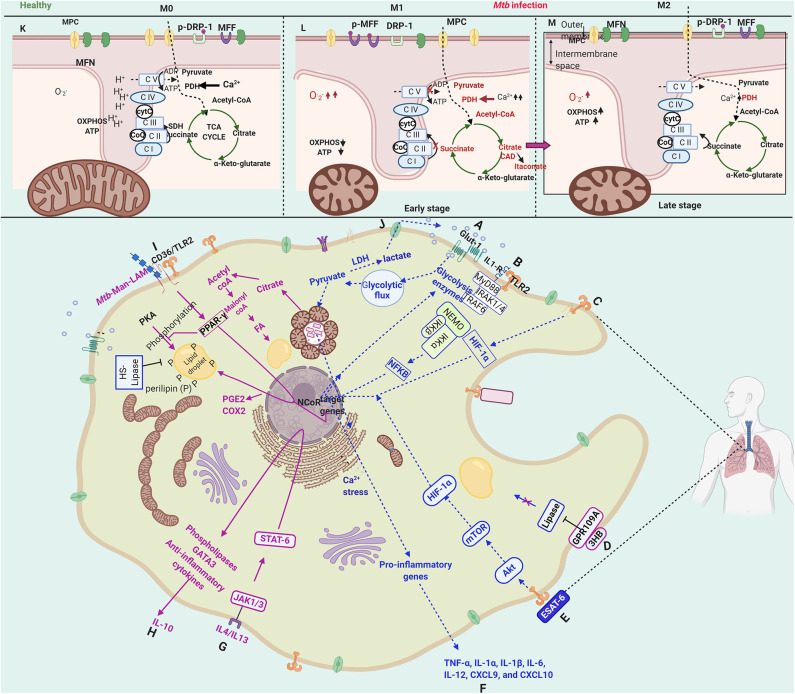
Schematic representation of immunometabolic and bioenergetic changes in a macrophage infected with *Mycobacterium tuberculosis*. Upon *Mtb* infection, host macrophage is activated by sensing several bacterial proteins, lipids, carbohydrates through various receptors (such as IL-1R, TLR2, CD36). The macrophage activation leads to a multitude of hostile cellular, immunological, metabolic, bioenergetic changes toward bacterial clearance, denoted as M1. The M1 phase is characterized by an increased glycolytic flux **(A)**, known as the Warburg-like effect. The changes during this phase are mainly brought about by NFkB **(B)** and HIF-1α **(C)**, which induce the transcription of glycolytic enzymes. At the immunological front, pro-inflammatory cytokines and chemokines, including IL-1α/β, IL-6, IL-12, CXCL9, and CCL10, are secreted **(F)**, which directly or indirectly contribute to bacterial clearance. During this phase, ROS is generated that is channeled into phagosomes to impose oxidative stress and as a signal for various cell death modalities. However, in the M2 phase, *Mtb* takes control over the cell and modulates the immunological, metabolic, and bioenergetic processes with the induction of PPAR-γ **(I)**, PGC-1α and STAT-6 **(G)**. They contribute to dampening the pro-inflammatory responses by secretion of IL-10 and alter lipid metabolism **(H)**, favoring carbon rerouting through fatty acids and accumulation of fatty acids in the form of lipid droplets **(D,I)**, converting the macrophages to what are known as “foamy macrophages.” PPAR-γ also contributes to the synthesis of PGE2 and COX2 **(I)**, which inhibit apoptosis and divert the cell toward necrosis, a cell death mode essential for the dissemination of *Mtb*. ESAT-6 signals to converts the M1 phase to M2 by reinforcing the GPR109A pathway, which has anti-lipase activity **(E)**.The pathways related to M1 are shown in blue dotted lines, and those related to M2 are shown in solid pink lines. The upper panel **(K–M)** shows the distinctions in the internal membrane structure of mitochondria and the changes in bioenergetic metabolism with accumulated metabolites and up-regulated enzymes in red. The metabolic changes take a turn through increased glycolytic flux **(A)** and decreased OXPHOS in M1 as compared to M0 compare upper panels **(K,L)**. The TCA cycle breaks at two points contributing to the accumulation of succinate, itaconate, and α-ketoglutarate **(K)**, which display microbicidal properties at various points. The TCA cycle is reinstated in M2, where *Mtb* takes control of host macrophage by modulating host defenses through altered energy metabolism. NADP oxidase subunits occur more abundantly upon infection in the mitochondrial membranes. Besides, the Ca^2+^ dynamics play a key role in the homeostasis of mitochondria; an increased Ca^2+^ level causes mitochondrial fragmentation, which co-relates with the increased presence of fission proteins (DRP1/MFF) and decreased presence of fusion proteins (MFN1/2). Bioenergetic processes are altered as compared to M0 with decreased OXPHOS, ATP **(L)** that correlates with the decreased number of ATP synthase subunits. The figure has been made using the Biorender app.

Resting macrophages are activated upon interaction with either bacterial ligands or cytokines. Depending on the immunological nature of cytokines secreted by the activated macrophage, the they are referred to as M1 (classical activation) that secretes pro-inflammatory cytokines or M2 polarised (alternate activation) that secretes regulatory cytokines. Interestingly, these polarized states are strongly co-related with distinct metabolic states. The early phase (2–8 h) of *Mtb* infection of macrophages, which shows a transcriptional profile characteristic of M1 polarization, is also marked with a metabolic switch from oxidative phosphorylation to aerobic glycolysis (Appelberg et al., 2015). A schematic overview of the immunometabolic and bioenergetic changes in *Mtb* infected macrophages has been depicted in Figure 3. The critical factors that initiate M1 polarization are the hypoxia-inducible factor-α (HIF-1α) (Appelberg et al., 2015) (Path C, Figure 3) and NFκB (Fong et al., 2008) (Path B, Figure 3) that induce the glycolysis genes and repress TCA cycle enzymes. *Mtb* infection causes a bi-phasic immunometabolic response with M1 during the early infection phase (2–8 h) and switching into M2 later during infection. The M1 state co-relates with high glycolytic flux and a decreased OXPHOS, whereas M2 state co-relates with decreased glycolysis and an OXPHOS (Mantovani et al., 2004; Lachmandas et al., 2016a,b; Shi et al., 2019). M1 macrophages are characterized by bactericidal activity *in vitro*, while M2 macrophages inhibit these effects and tend to support mycobacterial persistence through anti-inflammatory signaling (Shi et al., 2016).

HIF-1α is shown to regulate the expression of several glycolytic enzymes independent of IFN-γ including lactate dehydrogenase-A (LDH-A) in *Mtb*-infected macrophages. It induces the expression of LDH-A that prevents the accumulation of pyruvate, which otherwise may serve as a carbon source better than glucose for intracellular mycobacteria (Osada-Oka et al., 2019). The M1 phase induces the expression of several genes through IFN-γ such as IL-1β, IL-12, TNF-α, and iNOS/NOS2 (inducible NO synthase), the products of which result in bacterial clearance. For instance, TNF-α signaling results in apoptosis; IL-1β signaling results in ROS generation, IL-12 is a determiner of protective immunity against *Mtb* (Mata-Espinosa et al., 2019) which is produced by macrophages upon interaction with *Mtb* antigens. The secreted IL-12 signals through the JAK-STAT pathway to induce IFN-γ that in turn switches on various antimicrobial defenses. As compared to cytokine activated M1 macrophage, *Mtb* induces a more robust pro-inflammatory response, around 10-fold higher. The M1 phase is followed by the M2 phase, which depending on the nature of the activator and response, is sub-categorized into M2a, M2b, and M2c. *Mtb* infection induces the M2b type response. The transcriptional landscape of *Mtb* infected macrophages as compared to cytokine activated macrophages through the very early to late infection stages reveals that among the various genes induced upon *Mtb* infection, host inflammation and protection were upregulated more robustly (>10-fold) than M1 (IFN-γ activated) or M2 (IL4/IL13 activated) activated macrophages. Around 25% of the genes were differentially regulated as compared to resting macrophages (M0). The transcription profile was staggered with apoptosis/cell death regulators activated very early (before 4 h), followed by cytokines (4–24 h), chromatin rearrangement (12–24 h), and then regulatory events (24–48 h). The transcriptomic profile demonstrates a 3-phase gene expression. Around 75% of differentially expressed genes were like M1 macrophages. Several receptors, signaling molecules, kinases, cytokines, chemokines, and transcription factor (NFκB), were differentially regulated upon *Mtb* infection as compared to cytokine activated M1 or M2 macrophages (Roy et al., 2018). The *Mtb* secreted factor, ESAT-6, is reported to induce M1 polarization, which is mediated by the AKT/mTOR pathway in a TLR2 dependent manner (Path E, Figure 3). The inhibition of the AKT/mTOR pathway results in inhibition of cellular responses to *Mtb* (Lachmandas et al., 2016a). Besides, the GLUT-1 transporters are translocated from cytosol to the plasma membrane that enhances glucose import and resulting in a glycolytic flux (Path A, Figure 3). Further, PDH subunit genes are down-regulated that block the conversion of pyruvate to Acetyl-CoA (Shi et al., 2015). The shift to aerobic glycolysis is a critical parameter in deciding the fate of infection. Inhibition of this shift reduced IL-1β and decreased transcription of prostaglandin-endoperoxide synthase-2 (PTGS2), a key enzyme in prostaglandin biosynthesis and involved in inflammation. Simultaneously, it results in increased levels of anti-inflammatory IL-10 and increased intracellular bacillary survival (Gleeson et al., 2016). Blockade or absence of IL-1R negated the impact of aerobic glycolysis on intracellular bacillary survival, demonstrating that infection-induced glycolysis limits *Mtb* survival in macrophages through the induction of IL-1β.

On the other hand, inside the mitochondria, the TCA cycle suffers breaks resulting in the accumulation of intermediates including citrate, succinate, and α-ketoglutaric acid (Shin et al., 2011) as a consequence of transcriptional repression and also nitrosation of isocitrate dehydrogenase (IDH) (Tannahill et al., 2013; Bailey et al., 2019) (Upper panel, Path L, Figure 3). The accumulated citrate is transported out of mitochondria and is converted to Acetyl-CoA and further to Malonyl-CoA. An induction of CAD (cis-aconitate decarboxylase) activity results in the synthesis and accumulation of itaconate, an antimicrobial metabolite that inhibits *Mtb* isocitrate lyase and host succinate dehydrogenase also participates in pro-inflammatory signaling (Ryan and O'neill, 2020). Succinate acts at different levels toward bacterial clearance by inhibiting prolyl hydroxylase and stabilizes HIF-1α; maintaining pro-inflammatory signals. Secreted succinate binds to the succinate receptor to strengthen pro-inflammatory responses and further chemotaxis. α-ketoglutaric acid activates prolyl hydroxylases in contrast to succinate and destabilizes HIF-1α (Zdzisińska et al., 2017). In cancer cells, α-KG and its derivatives, when added to the medium, caused hypoxia-mediated cell death (Rzeski et al., 2012). Acetyl-CoA acetylates tubulin to activate anti-inflammatory IL-10, whereas malonyl-CoA malonylates lysine residues of enzymes of the glycolytic pathway and lipid synthesis (discussed extensively in Ryan and O'neill, 2020). As a consequence, the imbalance of glycolysis and TCA cycle results in the accumulation of DHAP and Acetyl-CoA that serve as substrates for the synthesis of lipids, that possibly results in the “foamy phenotype” discussed in a later section. The secreted ketone body, D-3-hydroxybutyrate (3HB) activates anti-lipolytic G-protein coupled receptor GPR109A and results in the inhibition of perilipin and Hormone Sensitive Lipase (HSL), further arresting lipid catabolism (Path D, Figure 3). The ketone bodies, 3HB, accumulated due to increased glycolysis induced by ESAT-6 reinforce this pathway (Singh et al., 2012). It is noteworthy that the M1 phase being a pro-inflammatory state churns out few metabolites that display anti-inflammatory activities that are probably required to regulate excessive inflammation and protect the cell.

#### M2 Phase: The Overturn of Host Defense Pathways by Mycobacterium Tuberculosis

Toward the late phase of infection (24–48 h post-infection), there is yet another metabolic switch, from glycolysis to oxidative phosphorylation, referred to as the M2 phase or alternate activation. The M1-M2 switch is brought about by PGC1β, PPARs, and STAT-6, which induce the expression of anti-inflammatory genes (Odegaard and Chawla, 2011). PPAR-γ, a lipid activated nuclear receptor and involved in the transition from M1 to M2 state, causes increased lipid droplet formation and down-regulates macrophage immune responses. It is probably an *Mtb* induced host switch to mycobacterial virulence and provides a host immune escape mechanism (Almeida et al., 2012). PPAR-γ is induced by *Mtb* through Man-LAM that further induces IL-8 and Cox-2 (Rajaram et al., 2010) and regulates lipid metabolism. The PPAR-γ expression is dependent on TLR2/CD36 receptor binding and subsequent signaling (Mahajan et al., 2012). *PPAR-*γ knockout mouse, when challenged with *Mtb* infection, demonstrated protection against the disease (Leemans et al., 2001). IL-4 and IL-13, the M2 markers, secreted during this phase, activate STAT6 that further activates phospholipases and GATA3 and, finally, IL-10 (Viola et al., 2019) (Path G, H, Figure 3). The switch to M2 phase, marked by the reduction in glycolysis of host macrophages was shown to be mediated by miR-21 that targeted Phosphofructokinase Muscle (PFK-M) that in turn reduced pro-inflammatory cytokines such as IL-1β and promoted bacterial growth (Hackett et al., 2020) with an intact TCA cycle. The M2 phase is also marked by glycosylation of mannose and lectin receptors, increased fatty acid oxidation, increased expression of CD36, a lipid scavenger receptor, and increased liposomal lipolysis and glutaminolysis that produces α-ketoglutarate, which causes epigenetic changes in the nuclear DNA and alters host gene expression. Increased fatty acid oxidation provides the primary carbon source for *Mtb* replication, as reviewed in Wilson et al. (2019). Depletion of M2 macrophages reduced bacterial burden by increasing the Th1 response; however, the depletion of M1 macrophages increased bacterial burden (Leemans et al., 2001). The proportion of M2 activated vs. M1 activated host cells determine the progress of the disease (Wilson et al., 2019).

### Critical Links Between Bioenergetics, Macrophage Response, and the Fate of *Mycobacterium tuberculosis* Infection

The M1 phase is essentially a host defense phase wherein it imposes pro-inflammatory and antimicrobial responses on the bacteria toward bacterial clearance as described. Avirulent mycobacteria succumb to such responses, but whereas the virulent mycobacteria have evolved multiple regulatory controls which overturn these attacks and enter an anti-inflammatory state, the M2 phase. The switch of an activated macrophage phase M1 to a restrained M2 phase that supports *Mtb* growth and further dissemination majorly involves cAMP signaling, redox homeostasis, and altered lipid metabolism (Path D, I, Figure 3).

#### Redox Homeostasis (Role of CO, NO, H_2_S)

The ROS and RNS generated in host mitochondria change the redox status (Farhana et al., 2010). Redox homeostasis plays an essential role in the functioning of mitochondria, which is maintained by several enzymes (superoxide dismutase, peroxidases, peroxiredoxins, catalase, and glutathione peroxidase) and non-enzymatic molecular species (Glutathione, several redox couples such as NAD+/NADH; FAD+/FADH2; NO, CO, H_2_S). Among the redox gasotransmitters such as NO, CO, and H_2_S, NO and CO have been well-studied in the context of cellular respiration. While NO and CO contribute to oxidative stress, H_2_S contributes to reductive homeostasis and reverses the redox changes mediated by NO. NO secretion is a well-known antimicrobial defense utilized by macrophages, to which *Mtb* is exposed in the phagosome. NO has a reasonably strong oxidizing potential and inhibits respiratory complex IV (Cytochrome C oxidase) (Brown, 1995), but whereas CO competes with O_2_ and binds to cytochrome C Oxidase with affinity almost equal to 1 (Queiroga et al., 2012). It is a part of the pro-inflammatory response by IFN-γ signaling that induces the expression of iNOS amongst others that produces NO using L-arginine. iNOS deficient mice show greater dissemination and succumb to death much earlier as compared to wild type mice (Macmicking et al., 1997). Both NO and CO stimulate dormancy regulon of *Mtb*. CO is induced by *Mtb* infection and produced by the enzymatic activity of heme oxygenase, HO-1 (Kumar et al., 2008).

The role of H_2_S that had remained unknown is now emerging as a crucial regulator of mitochondrial functions and can be considered to affect macrophage polarization. H_2_S causes a reversal of NO and CO-mediated inhibition of mitochondrial respiration. Macrophages generate H_2_S by using two enzymes, cystathionine β-synthase (CBS) and cystathionine γ-lyase (CSE). *Mtb* infection induces a 34-fold increase in CBS (Saini et al., 2020). Gene knockout studies using CSE^−/−^ (Rahman et al., 2020) and CBS ^+/−^ (Saini et al., 2020) mice revealed H_2_S promotes tuberculosis progression by suppressing carbon metabolism and dysregulation of the immune response (Rahman et al., 2020) and thus reverses Warburg-like effect of M1 macrophages. H_2_S reverses NO-mediated inhibition of respiration as well as regulates genes of sulfur and copper metabolism and the DOS regulon. At lower concentration range (5–25 μM) of H_2_S stimulates OXPHOS, cellular bioenergetics and stimulates *Mtb* growth, respiration, and pathogenesis whereas a higher concentration range (50–100 μM) results in opposite effects (Fu et al., 2012; Saini et al., 2020).

#### Lipid Droplet and Foamy Macrophages

The formation of lipid bodies/lipid droplets is a characteristic of macrophages upon infection with *Mtb*. Lipid droplets are lipid accumulations covered with a phospholipid monolayer protected from hydrolysis by the protein, perilipin, and also harbor several proteins of lipid metabolism. They interact with various organelles, including mitochondria, ER, and lysosomes (Valm et al., 2017). Lipid body accumulation within macrophages leading to foamy macrophages (FM) has been shown both in clinical and experimental mycobacterial infections, which can be mediated by IL-10 that enhances enzyme acyl CoA: cholesterol acyltransferase (ACAT) (Genoula et al., 2018). FMs are classically fat-laden M2 macrophages containing low-density lipoproteins (LDL), which are a clear case of altered metabolism. Much of the success of *Mtb* could be attributed to its lipid envelope that changes with space and time and promotes the induction of FM (Kim et al., 2010; Dulberger et al., 2020). Alteration in lipid metabolism coincides with M2 activation and *Mtb* control over the host cell.

The *Mtb* secreted multi-tasking protein ESAT-6, *via* induction of GLUT-1 mediated enhanced glucose uptake by macrophages, causes metabolic flux perturbations in the glycolytic pathway through accumulated DHAP and AcCoA, steering lipid accumulation toward FM differentiation. The protein-protein interactions between ESAT-6 and host Enolase1 and Phosphoglycerate kinase1 has been suggested that encourages further investigation (Singh et al., 2015). Another determinant of lipid metabolism that maintains lipid droplets is mi-R33, which is induced by *Mtb* that down-regulated autophagy and lipid catabolism (Ouimet et al., 2016). The increased fatty acids (TAG) are accumulated in the form of lipid bodies (LB), and the macrophages containing these LBs are termed foamy macrophages (FM) (Daniel et al., 2011). During *Mtb* infection, levels of cAMP are gradually reduced, which inhibits lipolysis and maintenance of LBs. *Mtb* induces perilipin synthesis, on the one hand, and the other hand, inhibits its PKA dependent phosphorylation to inhibit lipolysis (Singh et al., 2012). Under hypoxia conditions (granuloma), the *Mtb* laden phagosomes surround LBs, engulfed into lipid droplets. They, therefore, enjoy a luxurious and secure environment safeguarded against all the odds of the hostile environment are also resistant to the action of several drugs (Peyron et al., 2008). *Mtb* releases different kinds of lipid moieties, such as (TMM, TDM), phosphatidylinositol mannosidase (PIMs), LAM, and PGL-1, which are carried across to the bystander cells in a granuloma by exocytosis (Rhoades et al., 2003). However, the lipid accumulation in infected macrophages has been debated as induced both by the host to contain mycobacteria (Ulrichs and Kaufmann, 2006) or induced by mycobacteria for its nutritional requirements (Davis and Ramakrishnan, 2009). In a recent study (Knight et al., 2018), Knight et al. demonstrate that lipid bodies are induced by host IFN-γ signaling dependent on HIF-1α wherein the macrophage lipids are directed to lipid bodies and are unusable for mycobacterial consumption. In the study, they also tracked the mobilization of labeled fatty acid BODIPY FL C16 and observed that the labeled fatty acids get accumulated in the bacteria and increases as a function of time (2–8 h) even before lipid bodies are formed in the resting BMDM. However, the accumulation was not significant in IFN-γ activated BMDM, although the host macrophages had adequate lipid bodies. HIF-1α mediates IFN-γ dependent lipid droplet formation, and HIF-1α knockout accumulates very little lipid droplets. Further, proteomics of lipid droplets in THP-1 macrophages reveals that upon *Mtb* infection, around 57 proteins are upregulated and 29 proteins are down regulated. These proteins include those of protein synthesis, lipid metabolism and vescicle trafiicking, which were thought to promote increased mobilization of lipids. The role of secreted proteins in the modulation of the proteome of lipid droplets, for instance, the role of LipY has been discussed (Menon et al., 2019). Given the opposing schools of thought that the lipid droplets are host induced or *Mtb*-induced, and the distinct signaling events in both cases, it appears that the lipid droplets are complex and dynamic entities that are modulated by *Mtb* by rewiring the immunometabolism of the host with IFN-γ driven lipid body in M1 state to IL-10 driven lipid droplets in M2 state that is accessible to *Mtb* following a change in proteomic composition. The significance of using fatty acids from different sources will be interesting to investigate further.

Another facet of mitochondrial metabolism is the utilization of various carbon sources for ATP generation. *Mtb* is known to use host triacylglycerol and acquires a dormancy-like state in lipid-rich macrophages (Daniel et al., 2011). This led to the common belief that *Mtb* uses the endogenous fatty acid sources for its persistence inside macrophages. A more in-depth investigation using three substrates, glucose, glutamine and fatty acids, showed that *Mtb* infected hMDMs, use dominantly exogenous fatty acids rather than endogenous fatty acids under stress. hMDMs infected with *M. bovis-*BCG, the attenuated strain, utilizes both exogenous and endogenous sources of fatty acids, but unlike virulent *Mtb* strain, predominantly used endogenous fatty acid oxidation to meet energy requirement during stress (Cumming et al., 2018). These observations helped to dissect the finer steps of lipid utilization by *Mtb* during infection.

#### The Second Messenger cAMP

One other critical metabolite that is upregulated during infection of *Mtb* is cAMP (Bai et al., 2009), which is crucial to *Mtb* signaling and pathogenesis. The cAMP is a universal second messenger and signals both in prokaryotes and eukaryotes. These messengers also influence host gene expression and cause activation of protein kinase-A, which eventually activates the transcription factor CREB and alters host immune responses. the cAMP also regulates propionate metabolism and, therefore, cholesterol assimilation (Vanderven et al., 2015). cAMP produced by *Mtb* interferes with cAMP signaling in host cells and is required for bacterial virulence. The gene knockout of *Mtb* Rv0386, which encodes adenylate cyclase, resulted in a decrease in immunopathology and bacterial survival (Agarwal et al., 2009). Other adenylate cyclases influencing mycobacterial virulence include Rv2212 (Shleeva et al., 2017) and Rv1675c (Smith et al., 2017). Unlike cAMP, c-di-AMP, and c-di-GMP serve as an alarm signal for the mammalian immune system (Karaolis et al., 2007). *Mtb*-produced cAMP and c-di-AMP affect the host cell signaling during infection. The role of c-di-GMP in TB pathogenesis is not well understood. By chemically targeting *Mtb* adenyl cyclases, the replication of *Mtb* could be controlled. The small molecule V-58 activates *Mtb-*Adenyl cyclase Rv1625c to produce cAMP, which inhibits *Mtb* growth on cholesterol and propionate and also interferes with host signaling by regulating TNF-α (Johnson et al., 2017). Therefore, the small molecule V-58 specifically inhibits *Mtb* and not *M. smegmatis* conforming to physiological sources of nutrients.

## Conclusive Remarks

Mitochondria play a critical role in cellular functions of varied ontogeny and are often targeted by many pathogenic viruses and intracellular bacteria. The outcome of host-pathogen interactions in tuberculosis is dynamic and depends on several variables such as time point of evaluation of host response, the ontogeny of cell type, and *in-vitro* vs. *in-vivo* studies. Therefore, the smooth integration of the available data toward understanding the mitochondrial control of *Mtb* through various approaches may not be possible. We propose that a set of guidelines and definitions be made for *Mtb* studies so that there remains a uniformity in what is called an early or a late phase, how much infection burden (MoI) and duration of infection to be followed to call it a mimic of chronic infection, what infection burden is defined closest to physiological conditions, what *in-vitro* stresses can be taken as closest to intra-phagosomal microbicidal conditions, and so on so that experiments across the laboratories can be compared and correlated. However, the diverse studies contribute to our understanding of the discrepancy in their responses to infection. As demonstrated by Huang et al. (2018), the ontogeny of the macrophages determines the metabolic state and, thus, the diverse responses to infection. The early macrophage pro-inflammatory responses are overturned by *Mtb* 24–48 h post-infection in the biphasic response (Shi et al., 2019).

It is largely understood that *Mtb* infection skews the pro-inflammatory responses of the host macrophage to anti-inflammatory, which coincides with a shift in energy metabolism from glycolysis to OXPHOS with fatty acids taking center stage. To the utmost surprise of immunologists, the Krebs cycle has emerged as the central regulator of immune cell effector functions wherein, accumulation of metabolites including succinate, citrate, and itaconate occurs that have an antimicrobial effect Ryan and O'neill, 2020. The role of *Mtb* virulence in this metabolic rewiring of host mitochondria becomes clear when compared to infection with non-pathogenic mycobacterial strains, wherein glycolytic flux is much greater (Cumming et al., 2018) and reflects the dominance of host induced pro-inflammatory response. The OXPHOS status differs during the infection process, taking a backstage in early infection, referred to as M1, and reinstated later in infection as discussed elaborately. The OXPHOS status also co-relates with mitochondrial morphology. In the M1 state, where there is decelerated OXPHOS, the mitochondria are discrete without any networks, but whereas in M2 (several sub-states occur in various cellular contexts), the networks are partly restored where OXPHOS is active (Li et al., 2020).

The role of metabolites in cell death pathways is unfamiliar. In a recent review (Ryan and O'neill, 2020), the role of succinate, citrate, and itaconate in pro-inflammatory signaling was discussed. However, the mechanisms of metabolic regulation driving this process are not quite clear. Nevertheless, the significance of ATP levels in triggering the type of host cell death is known. *Mtb* harbors factors that can either induce cell death or inhibit circumstantially. For instance, Cpn60.2 is an abundant chaperone of *Mtb* that gets dissociated from cell surface upon phagosomal entry and finally reaches mitochondria, where it interacts with mitochondrial mortalin promoting cell survival (Joseph et al., 2017). The host response to infection is much weakened in other metabolic disorders, such as diabetes, wherein the mitochondria are already compromised. *Mtb* infection in such a state of the cell causes more severe derailment of host mitochondria. Macrophages pre-treated with sub-pathological levels of cholesterol, simulating borderline dyslipidemia, infected with *Mtb*, showed more pronounced alterations in mitochondrial structures (Asalla et al., 2017). Besides, non-pathogenic mycobacteria *M. bovis-*BCG also cause an increase in intermediary and lipid metabolism that supports co-infections, as observed in the case of HIV-mycobacteria, where such alterations in metabolism correlated with both higher viral titers (Ganji et al., 2016a), and reduced clearance of mycobacteria (Ganji et al., 2016b).

## Future Perspectives

In the era of drug resistance, host-directed therapy is emerging as a robust adjunct therapy that could help in reducing host pathology and strengthens host immune signaling such as by restoring mitochondrial health. A small molecule, M1 that restores mitochondrial biogenesis balance, could revert the detrimental impact of mycobacterial infection and improved the clearance of *Mtb* by macrophages (Asalla et al., 2017), strongly pointing to the possibility of improving mitochondrial health as a host-directed therapy. Vitamin D reverses PPAR-γ mediated adipogenic effects and therefore curtails *Mtb* growth (Salamon et al., 2014). The MMT inhibitors cyclosporin A corticosteroids (Grab et al., 2019) also offer targets for host-directed therapy. Many of the pro-drug conversions depend on redox status; an understanding of redox homeostasis, which is currently less explored, and understanding the factors involved therein, would help in increasing the efficacy of the drugs. Targeting host synthesized H_2_S that promotes *Mtb* growth and survival can contribute toward control of TB (Rahman et al., 2020). A list of potential host therapy targets based on autophagy and immunometabolism are listed in Table 2, some of which are in clinical trials. Drug repurposing involving the restoration of mitochondrial health could be promising candidates for further investigation.

**Table 2:** Host directed therapy targeting mitochondrial functions toward tuberculosis management.

**S.no**	**Inhibitor/drug**	**Target**	**Remarks**	**References**
1	Metformin	Phosphorylates mTOR and p70S6k	Autophagy interrupts the mitochondrial respiratory chain and induces production of ROS	(Naicker et al., 2020)
2	M1	Mitochondrial fusion	Restores Mitochondrial functions	(Asalla et al., 2017)
3	Cystamine/cysteamine	Transglutaminase	increasing glutathione and L-cysteine level	(Palucci et al., 2019)
4	Statins	HMG CoA reductase	Autophagy Block lipid accumulation	(Palucci and Delogu, 2018)
5	Alisporivir	PTP inhibitor cyclophilin D	Inhibits ROS Necrosis is inhibited without affecting bacterial clearance In phase III clinical trials	(Šileikyte and Forte, 2016))
6	VitD3	Stimulates vitamin D receptor and induces cathelicidin expression as well as Atg5 and Beclin-1	Autophagy reverses PPAR-γ mediated adipogenic effects	(Palucci and Delogu, 2018)
7	Aspirin ibuprofen zileuton	Block eicosanoids	Modulates inflammation through classical COX-dependent inhibition of prostaglandins	(Tobin, 2015)
8	H-89/ETB089	cAMP-dependent PKA inhibitor	Enhance Autophagy	(Kuijl et al., 2007)
9	Pioglitazone, rosiglitazone, and treprostinil	PPAR-γ	Regulate genes of glucose and lipid metabolism and decrease triglycerides and increase insulin uptake	(Rask-Andersen et al., 2014)
10	Mepenzolate bromide	G-protein-coupled receptor GPR109A pathway	bacterial burden was reduced in cell culture and in mouse *in vivo* models	(Singh et al., 2012)
11	Rapamycin	mTOR	Enhances autophagy	(Singh and Subbian, 2018)
12	Cyclosporine corticosteroids	MMT inhibitors	Inhibit necrosis	(Gan et al., 2005; Grab et al., 2019)

## Author Contributions

KM and SB conceived the structure and content, revised the manuscript, and made the final version. KM, JM, and SB wrote the first draft. GV added extra information and helped with critical reading and organization. All the authors have read and approved the final manuscript.

## Conflict of Interest

The authors declare that the research was conducted in the absence of any commercial or financial relationships that could be construed as a potential conflict of interest.

## Abbreviations

3HB: D-3-Hydroxybutyrate
ADP: Adenosine Di Phosphate
AEC: Adenylate Energy Charge
AKT-1: RAC-Apha Serine/Threonine-Protein Kinase
AMP: Adenosine Mono Phosphate
AMPK 5’: Adenosine Mono Phosphate-Activated Protein Kinase
ATF-3: ActivatingTranscription Factor 3
ATG-12: Autophagy Related Protein 12
ATG-3: Autophagy Related Protein 3
ATP: Adenosine Tri Phosphate
BCG: Bacillus Calmette Guerin
BCL2: B-Cell Leukemia/Lymphoma 2
BCL-XL: B-Cell Lymphoma-Extra Large
CAD: *cis*-aconitate decarboxylase
cAMP, 3: 5- Cyclic AMP
CBS: Cystathionine β-Synthase
Cpn60. 2: Chaperonin 60.2
CREB: cAMP Response Element-Binding Protein
CSE: Cystathionine γ-Lyase
DAT: Di-acyltrehalose
DG70: Biphenyl Benzamide GSK1733953A (An inhibitor)
DHAP: Di-hydroxy acetone phosphate
DNA: Deoxy Ribonucleic Acid
DRP-1: Dynamin-Related Protein-1
EGT: Ergothioneine
Erg_ox_/Erg_red_: Ergothioneine redox couple
ESAT-6: Early Secretory Antigenic Target-6
ETC: Electron Transport Chain
FAD: Flavin Adenine Dinucleotide
FADH_2_: Flavin Adenine Dinucleotide reduced form
FASII Pathway: Fatty Acyl Synthesis Type II Pathway
FAS: Fatty Acyl Synthase
Fe-S Custer: Iron Sulfur Cluster
GLUT-1: Glucose Transporter 1
GroEL2: 60 KDa Chaperonin
H_2_S: Hydrogen Sulfide
HBHA: Heparin Binding Heme Agglutinin Protein
Hif-1α: Hypoxia Inducible Factor Alpha
HIV: Human Immunodeficiency Virus
HIV-BCG: Human Immunodeficiency Virus-Bacillus Calmette Guerin
HSP: Heat Shock Protein
IDH: Isocitrate Dehydrogenase
IFN-γ: Interferon Gamma
IL-10: Interleukin 10
IL-1β -: Interleukin 1 Beta
IL-4: Interleukin 4
IL-6: Interleukin 6
IMM: Inner Mitochondrial Membrane
IP3: Inositol Triphosphate
JNK: C-Jun- N-Terminal Kinase
Lpqh: Lipoprotein LpqH
LprG: Lipoarabinomannan carrier protein LprG or Antigen p27
LPS: Lipo-polysaccharide
*M. bovis-*BCG: *Mycobacterium Bovis* Bacillus Calmette Guerin
MCP-1: Monocyte Chemoattractant Protein-1
MCS: Membrane Contact Site
MFF: Mitochondrial Fission Factor
MFN1: Mitofusin 1
MFN2: Mitofusin 2
miR-21: microRNA 21
MMP: Mitochondrial Membrane Polarization
MMPT: Mitochondrial Membrane Permeability Transition
MMT: Methylcyclopentadienyl Manganese Tricarbonyl
MPTP: Mitochondrial Permeability Transition Pore
MSH: Mycothiol
*Mtb*: *Mycobacterium tuberculosis*
*Mtb*-H37Ra: *Mycobacterium tuberculosis* H37Ra
*Mtb-*H37Rv: *Mycobacterium tuberculosis* H37Rv
mTOR: Mammalian Target of Rapamycin
Mφ: Macrophages
NAD+: Nicotinamide Adenine Dinucleotide oxidized form
NADH: Nicotinamide Adenine Dinucleotide Dehydrogenase
NFκB: Nuclear Factor Kappa B
OPA1: Optic Atrophy 1 (A Mitochondrial Dynamin-Like Protein)
OXPHOS: Oxidative Phosphorylation
P47phox: NADPH oxidase cytosolic protein p47phox, also known as neutrophil cytosolic factor 1 (NCF1)
P67phox: NADPH oxidase cytosolic protein p67phox. it is also known as neutrophil cytosolic factor 2(NCF2)
PAT: Poly-acyltrehalose
PC: Phosphatidyl Choline
PDE: Phosphodiesterase
PDH: Pyruvate Dehydrogenase
PDIM: Phthiocerol dimycocerosates
PE: Phosphatidyl Ethanolamine
PE_PGRS33: Proline Glutamine Polymorphic GC Rich Sequence
PE-PPE: Proline-Glutamate Proline-Proline-Glutamate
PFK-M: Phosphofructokinase Muscle
PGC1β: Peroxisome Proliferator-Activated Receptor-Gamma Co-activator 1 beta
PINK1: Phosphatase and Tensin Homolog (PTEN) Induced Kinase 1
PMF: Proton Motive Force
PPARs: Peroxisome Proliferator-Activated Receptors
PS: Phosphatidyl Serine
Q203: Cytochrome *bc*1 complex inhibitor
RIPK3: Receptor-Interacting Serine/Threonine Protein Kinase 3
ROS: Reactive Oxygen Species
SL: Sulfolipids
STAT-6: Signal Transducer and Activator of Transcription 6
SOD 1: SuperOxide Dismutase 1
TB: Tuberculosis
TCA Cycle: Tri Carboxylic Acid Cycle
TDM: Trehalose Dimycolate
THP-1: Human monocytic leukemia cell line
TLR2: Toll-Like Receptor 2
TNFα: Tumor Necrosis Factor Alpha
Trxss/Trx(SH)_2_: Thioredoxin redox couple
Whib3: Redox- and pH-responsive transcriptional regulator WhiB3.
